# A neural algorithm for *Drosophila* linear and nonlinear decision-making

**DOI:** 10.1038/s41598-020-75628-y

**Published:** 2020-10-29

**Authors:** Feifei Zhao, Yi Zeng, Aike Guo, Haifeng Su, Bo Xu

**Affiliations:** 1 Research Center for Brain-inspired Intelligence, Institute of Automation, Chinese Academy of Sciences, Beijing, 100190 China; 2 Center for Excellence in Brain Science and Intelligence Technology, Chinese Academy of Sciences, Shanghai, 200031 China; 3 National Laboratory of Pattern Recognition, Institute of Automation, Chinese Academy of Sciences, Beijing, 100190 China; 4 University of Chinese Academy of Sciences, Beijing, 100049 China; 5 Institute of Neuroscience and State Key Laboratory of Neuroscience, Shanghai Institutes for Biological Sciences, Chinese Academy of Sciences, Shanghai, 200031 China; 6 State Key Laboratory of Brain and Cognitive Science, Institute of Biophysics, Chinese Academy of Sciences, Beijing, 100101 China; 7 Shanghai Institute of Microsystem and Information Technology, Shanghai, 200050 China; 8 Laboratory of Molecular Neural Biology, School of Life Sciences, Shanghai University, Shanghai, 200444 China

**Keywords:** Engineering, Computational neuroscience

## Abstract

It has been evidenced that vision-based decision-making in *Drosophila* consists of both simple perceptual (linear) decision and value-based (non-linear) decision. This paper proposes a general computational spiking neural network (SNN) model to explore how different brain areas are connected contributing to *Drosophila* linear and nonlinear decision-making behavior. First, our SNN model could successfully describe all the experimental findings in fly visual reinforcement learning and action selection among multiple conflicting choices as well. Second, our computational modeling shows that dopaminergic neuron-GABAergic neuron-mushroom body (DA-GABA-MB) works in a recurrent loop providing a key circuit for gain and gating mechanism of nonlinear decision making. Compared with existing models, our model shows more biologically plausible on the network design and working mechanism, and could amplify the small differences between two conflicting cues more clearly. Finally, based on the proposed model, the UAV could quickly learn to make clear-cut decisions among multiple visual choices and flexible reversal learning resembling to real fly. Compared with linear and uniform decision-making methods, the DA-GABA-MB mechanism helps UAV complete the decision-making task with fewer steps.

## Introduction

When confronted with two visual patterns and one of them is related to punishment, *Drosophila* could make appropriate choices to avoid punishment^1,2^. For example, facing an inverted-blue T is always companied with heat punishment, while facing upright-green T is safe in a visual flight simulator. Then, *Drosophila* could learn the association between visual input and punishment, and fly towards the safe pattern (upright-green T) even if the heat punishment is shut down. This is the simple perceptual decision-making task. To verify whether *Drosophila* could make choice between two conflicting cues, individual flies were conditioned to choose a flight direction in accordance with consistent color and shape cues, and then the decision was tested with the color and shape cues reversed following the training^3,4^. For example, during the training phase, upright-green T is trained to be safe, and inverted-blue T is with punishment. While during the choice phase, fly needs make choice between inverted-green T and upright-blue T. Which pattern should be chosen to avoid potential danger? Apparently, only simple perceptual decision-making circuit is not enough to make clear choice when confronting this shape-color dilemma. Neuroscientists found that with the help of the value-based circuit, *Drosophila* could make choice based on the saliency of two cues^3^. They measured the choice behavior of *Drosophila* by preference index (PI), which is defined by the ratio of the difference between times of *Drosophila* flying towards each pattern to total time^3^. Neuroscience experiments calculated the choice PI of both wild-type flies and mutant *mushroom body miniature(mbm)* flies with different saliency of color cues. Experimental results showed that the wild-type flies (with the value-based circuit) achieved a nonlinear (a sigmoid-fit) shape, while *mbm* flies (only simple perceptual circuit) achieved linear shape^3–5^. That is why the simple perceptual circuit is also called linear circuit, and the value-based circuit is also called non-linear circuit. Inspired by the neural mechanism of *Drosophila* linear and nonlinear decision-making circuit, this paper proposes a multi-brain area coordinated decision-making spiking neural network, and verifies the effectiveness of the proposed model on *Drosophila*-like decision-making tasks.

Although the linear and nonlinear circuits of *Drosophila* vision-based decision-making have achieved some biological evidence in^4,6^, it still has large space to improve the computational model of *Drosophila* vision-based decision-making. Hui Wei et al. designed a SNN to simulate *Drosophila* simple non-conflicting visual cues learning in the flight simulator. The learning process depends on spike timing dependent plasticity (STDP) updating weights between input neuron and output neuron. This learning process was implemented by inhibiting input neurons and activating output neurons when heat punishment happened^7^. Nevertheless, the network architecture and learning process need to be refined to capture important biological details and it could not solve the conflicting cues learning. Based on the decision-making neural circuit and learning process in^7^, Wei et al.^8^ further added conflicting cues learning during the choice phase. With the same training network, different input cues were entered into a certain decision-making unit with a probability determined by color-shape selection ability (linear or sigmoid selection ability) and current color intensity, then the decision-making unit triggered an action according to the learned stimulus-action association. Experimental results showed that using the linear-shaped color-shape selection curve can produce the linear-shaped PI, while using the sigmoid-shaped color-shape selection curve can produce the nonlinear-shaped PI. The difference between linear and nonlinear decision-making is based on the corresponding input color-shape selection curve, which shows little biological basis of the neural circuit. Kuijie Cai et al. built up a network model to elucidate the computational mechanism of *Drosophila* when confronted with shape-color dilemma^9^. The computational model could successfully describe the experimental findings in^3^. However, the model was based on some hypothetical connections and lacked some new-found decision-making mechanism in *Drosophila*. Wu et al. modeled the linear and nonlinear circuits based on some biological hypotheses. They aimed to account for *Drosophila* decision-making phenomena from systems level^10^. The effort mainly focused on comparing the effect of linear and nonlinear circuits on the conflicting visual cues learning. In order to simulate the nonlinear circuit, a raised dopamine level is used to enhance the lateral inhibition between decision-making units. To sum up, the present existing computational models have not taken the DA-GABA-MB mechanism (which is observed in *Drosophila* decision-making circuit^11,12^) into consideration. In this paper, we aim to design a dynamic decision-making SNN model, named as DrosDecMa, which is mostly based on the linear and nonlinear mechanisms of the *Drosophila* Decision-Making circuits.

Other studies focus on the construction of biologically inspired decision-making neural circuit. Zarin et al.^13^ generated a recurrent network model based on *Drosophila* larval muscle activity patterns and premotor/motor circuits to understand how the *Drosophila* generates forward and backward locomotion. Atiya et al.^14^ proposed a neural circuit model to monitor decision uncertainty, which was based on transient neural dynamics observed in animal and human studies. The proposed uncertainty-monitoring module can replicate several important observations found in experimental studies of change-of-mind. Inspired by the known anatomy and physiology of basal ganglia, which is the core brain area of the decision-making circuit in mammal, Héricé et al.^15^ developed a connectionist model from spiking neuron level based on a previous rate model approach. The model could provide predictions in accordance with observed experimental data on lesion tests, reversal learning, and extinction protocols tasks. Zhao et al.^16^ proposed a brain-inspired decision-making spiking neural network (BDM-SNN) to model the multi-brain areas coordinated decision-making circuit and applied it to decision-making tasks on intelligent agents. The proposed model has greater accuracy and is faster than traditional reinforcement learning method. Dezfouli et al.^17^ proposed a recurrent neural network to generate a flexible family of models that have sufficient capacity to represent the complex learning and decision-making strategies used by humans. On two-armed bandit task, the proposed method is better than baseline reinforcement-learning methods in terms of overall performance and its capacity to predict subjects’ choices. Kleinman et al.^18^ trained multi-area recurrent neural networks (RNNs) via gradient descent using backpropagation through time and Adam optimizer. Multi-area RNNs reproduced the decision-related dynamics observed in dorsal premotor cortex of monkeys on the Checkerboard task. These methods simulated the structure and function of decision circuit to a certain extent. The models either reproduced several observations found in neuroscience experimental studies or inspired a more intelligent decision-making algorithm.

The main contributions of this paper are as the following: (1) We model *Drosophila* vision-based linear and nonlinear decision-making system from spiking point neuron-level to brain areas-level and finally with similar behavior level outputs. (2) We design a conflict monitor as a switching rule between linear and nonlinear pathways to decide which one should be chosen when confronted with specific visual cues. (3) The DrosDecMa model presents similar abilities as the *Drosophila* trained in flight simulator on both non-conflicting and conflicting visual cues learning, and it could be applied to UAV for reinforcement learning and online reversal learning tasks. (4) We further discuss the significance of the DA-GABA-MB mechanism of the nonlinear circuit and apply it to the UAV decision-making tasks, such as the autonomous flying through window task and the obstacle avoidance task. Experimental results indicate that the DA-GABA-MB mechanism has good generalization on making clear-cut decisions among multiple conflicting choices.

## Results

### Simulation of *Drosophila* visual learning experiments

This section verifies the DrosDecMa model on *Drosophila* visual learning experiments which are almost the same as the visual learning experiments in^3^. The experiments are divided into two phases: the training phase and the choice phase. Each phase takes 2 s. Every time the model receives a pair of visual patterns, one is the current input pattern and the other is the inhibited pattern. The model’s output represents the chosen behavior. Behavior 1 means approaching the current pattern, and behavior 2 means flying towards another visual pattern (avoiding current pattern). Punishment only occurs in the training phase to modulate the network weight. We conduct several training-choice decision-making experiments to verify the effectiveness of our DrosDecMa model.

1 $$PI = {\raise0.7ex\hbox{${\left( {t_{1} - t_{2} } \right)}$} \!\mathord{\left/ {\vphantom {{\left( {t_{1} - t_{2} } \right)} {\left( {t_{1} + t_{2} } \right)}}}\right.\kern-\nulldelimiterspace} \!\lower0.7ex\hbox{${\left( {t_{1} + t_{2} } \right)}$}}$$

where *t*_1_ represents the total time of choosing behavior 1 and *t*_2_ represents the total time of choosing behavior 2.

### Learning single visual cue

**Figure 1:**
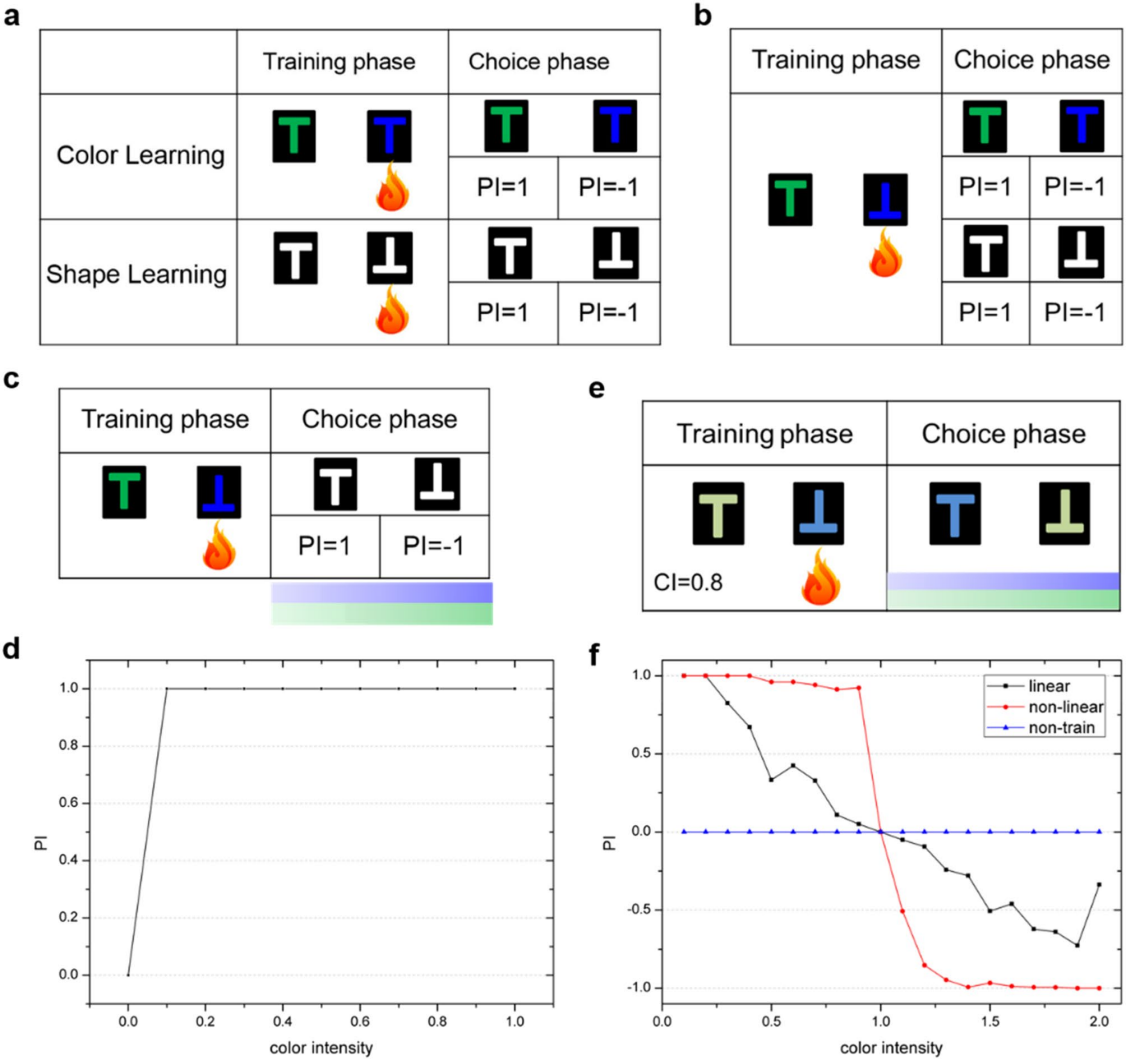
The simulation results of *Drosophila* visual learning experiments. (**a**) The training phase and the choice phase of single color or shape learning. The red fire mark represents heat punishment when facing a specific visual pattern. (**b**) The training phase and the choice phase of learning two visual cues. (**c**) After learning two visual cues, choice phase on shape learning over the range of CI from 0 to 1. (**d**) After learning two visual cues, choice phase on color learning over the range of CI from 0 to 1. X-axis represents the change of CI from 0 to 1, and y-axis represents the change of PI. (**e**) The training phase of learning two visual cues with *CI* = 0.8, and during the choice phase, the reversed visual cues are tested with CI value from 0.1 to 2. (**f**) For two conflicting cues learning, the experimental results on nonlinear decision-making network, linear decision-making network and non-trained network. X-axis represents the change of CI from 0.1 to 2, and y-axis represents the change of PI.

For single visual cue learning tasks, the color and shape cues are trained respectively, and the same patterns are used for training and choice phases, which are shown in Fig. 1a. Firstly, facing upright-green T is trained to be a safe state, while facing upright-blue T will get punishment. Then choices are made to evaluate the color learning ability. The numbers of choosing behavior 1 and 2 are counted during 2 s to calculate PI. PI is equal to 1 when facing upright-green T which means the model chooses approach behavior all the time, while PI is equal to − 1 when facing upright-blue T which means the model chooses avoidance behavior all the time. PI shows that the model could correctly choose safe behavior. This process is also acted on the shape learning task (upright-white T and inverted-white T), and the model could learn to prefer the safe shape.

### Learning two visual cues

For two visual cues learning tasks, the color and shape cues are trained simultaneously, and choices are made on a single cue, which is shown in Fig. 1b. Firstly, upright-green T is trained to be safe, and inverted-blue T is with punishment. Then we test whether the model has learned every single cue by making choice between upright-green T and upright-blue T (test color cue), and between upright-white T and inverted-white T (test shape cue). PI shows that the model approaches green color and avoids blue color for single color test, while approaches upright shape and avoids inverted shape for single shape test. As a result, the model has learned safe visual cues.

### The influence of color intensity (CI)

To test whether learning one cue is influenced by another cue, upright-green T and inverted-blue T (with heat punishment) are trained and choices are made on single color and shape patterns with the consideration of different color intensity (*CI* , with the range from 0 to 1). *CI* changes the value of color, such as $\left[ {0,10*CI,0,10,0} \right]$ represents the upright-green T with different color saliency (where $CI \in \left[ {0,0.1,0.2 \ldots 1} \right]$). Then we test shape learning by choice making between upright T and inverted T. The result is shown in Fig. 1c, where PI is equal to 1 when facing upright shape, and equal to − 1 when facing inverted shape. Besides, the value of PI is constantly equal to 1 and − 1 over the range of CI from 0 to 1. This indicates that shape learning is not influenced by the change of CI.

In addition, we test the color learning ability through choice making between upright-green T and upright-blue T. As shown in Fig. 1d, the visual input is upright-green T, and the color cue learning is influenced by CI. When *CI* < 0.1, PI is equal to 0. It indicates that the numbers of choosing behavior 1 and 2 are the same, and the model could not learn color cue. When *CI* ≥ 0.1, PI is constantly equal to 1. The turning point (*CI* = 0.1) indicates that only when the color cue is clear enough can the model learns color cue.

### Learning two conflicting visual cues

For two conflicting visual cues learning tasks, the nonlinear circuit is used to make clear-cut decisions. Here, we implement both linear and nonlinear circuits and analyze the decision-making ability of them, respectively. The training and choice phases are shown in Fig. 1e. Firstly, upright-green T is trained to be safe, and inverted-blue T is with punishment, with *CI* = 0.8. In the choice phase, we inverse the two cues by making choice between inverted-green T ($\left[ {0,10*CI,0,0,10} \right]$) and upright-blue T ($\left[ {0,0,10*CI,10,0} \right]$), and the visual input is upright-blue T. We test this shape-color dilemma choice experiment over the range of CI from 0.1 to 2 ($CI \in \left[ {0.1,0.2 \ldots 2} \right]$). Here, *CI* = 1 means the saliency of shape and color are the same, and *CI* > 1 means the color cue is more salient than shape cue, and *CI* < 1 means shape cue is more salient than color cue.

The experimental results on the nonlinear circuit and linear circuit are shown in Fig. 1f. The nonlinear circuit displays a sigmoid-shape curve, while the linear circuit displays a linear-shape curve. The blue line in Fig. 1f represents the PI value of the non-trained network, and PI is constantly equal to 0 because the non-trained network could not make choice between two conflicting cues. The black line represents the result of the linear decision-making network, where *PI* > 0 when *CI* < 1 and *PI* < 0 when *CI* > 1. *PI* > 0 indicates that the model almost approaches upright-blue T, while *PI* < 0 indicates that the model almost avoids upright-blue T, and the closer that the absolute value to 1, the more clear choice the model can make. Linear decision-making is ambiguous when the difference between saliency of two cues is slight, thus the curve is linear and the absolute value of PI is closer to 0. The red sigmoid-shape line in Fig. 1f is the result of nonlinear decision-making network. When *CI* < 1, *PI* > 0 indicates the model follows shape cue to approach upright-blue T. When *CI* > 1, the color cue is more salient than shape cue, then *PI* < 0 indicates that the model follows color cue to avoid upright-blue T. The result is consistent with the prediction. Comparing nonlinear curve with linear curve, it is obvious that nonlinear decision-making network could make clear-cut choice even if two cues have similar saliency. The reason is that the nonlinear circuit could amplify the slight difference between the two choices and then quickly make clear choice.

### Application of the DA-GABA-MB mechanism

To further explore the clear-cut decision-making ability of nonlinear circuit, we apply the DA-GABA-MB mechanism to the UAV multiple choices tasks in the natural scene. We conduct two types of UAV decision-making tasks: the UAV flying through the window and the UAV obstacle avoidance.

#### Application on the UAV flying through window task

**Figure 2:**
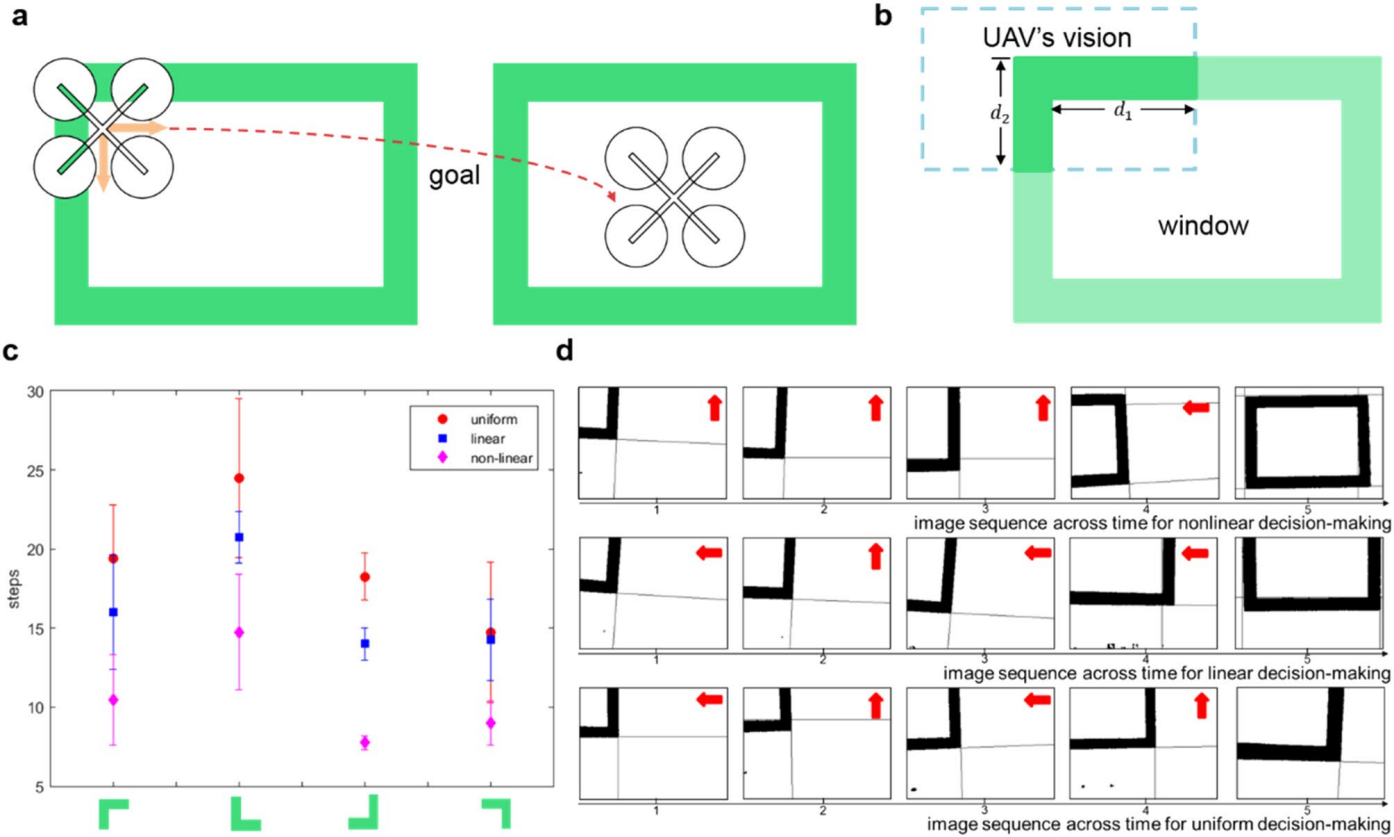
The experimental process and results on the UAV flying through window task. (**a**) The multiple choice problem of the UAV flying through window task. (**b**) Two conflicting cues of the UAV flying through window task. (**c**) The required steps from different initial states to goal by nonlinear, linear and uniform decision-making manners respectively. The X-axis represents the four initial states with two conflicting choices. (**d**) Some key image sequences with the same initial state and the similar input saliency by nonlinear, linear and uniform decision-making manners respectively. The red arrows represent the UAV’s flying direction.

The UAV is first situated in the corner of the window, then it learns the rules to fly close to the window’s center by the interaction with environment. The detailed reinforcement learning process is introduced in^19^. When the UAV is situated in the upper-left, upper-right, lower-left and lower-right corners of the window, there are two choices with reward. For example, when the UAV is situated in the upper-left corner as Fig. 2a shown, flying down and right are both able to reach the goal. Note that the required steps are different for different choices. How can the UAV make clear-cut decision between two choices? Here, we introduce the nonlinear decision-making mechanism to help the UAV make clear-cut decisions between two choices. The image of UAV’s vision is depicted in Fig. 2b, where the window’s width in the UAV’s vision is *d*_1_, and the window’s height in the UAV’s vision is *d*_2_. *d*_1_ and *d*_2_ represent the two conflicting cues, and the corresponding actions are flying along horizontal direction and vertical direction, respectively. Here, we test the required steps from initial states to goal state by nonlinear, linear, and uniform decision-making manners. For uniform decision-making manner, the probabilities of following horizontal cue and vertical cue are equal: *p*_1_ = *p*_2_ = 0.5. For linear and nonlinear circuits, the probability of following horizontal cue and vertical cue is $$p_{i} = {\raise0.7ex\hbox{${t_{i} }$} \!\mathord{\left/ {\vphantom {{t_{i} } {\mathop \sum \nolimits_{j}^{n} t_{j} }}}\right.\kern-\nulldelimiterspace} \!\lower0.7ex\hbox{${\mathop \sum \nolimits_{j}^{n} t_{j} }$}}$$, where *t*_*i*_ denotes the number of spikes of cue *i* in kenyon cells (KC) layer for nonlinear circuit (or input layer of central complex (CC) for linear circuit), *n* is the number of cues. We note that the steps from the same states to goal are similar for nonlinear, linear and uniform decision-making. The inputs *d*_1_ and *d*_2_ are normalized to suit the network’s input (from 1 to 20) of nonlinear and linear circuits.

Figure 2c shows the required steps from different initial states to goal by different decision-making manners. The required steps are the average of 16 trials for each manner. The nonlinear decision-making is the fastest manner, and the linear decision-making is slower than nonlinear decision-making, and the slowest one is uniform decision-making. Some key image sequences with the same initial state and the similar input saliency under different manners are shown in Fig. 2d. For nonlinear decision-making, the UAV always follows the more salient cue and quickly reaches the goal. For linear decision-making, there are also some steps following the shorter edge, so that the needed steps from initial state to another state is more than nonlinear decision-making. For uniform decision-making, the UAV is so indecisive between two choices that it needs more steps to reach another state. The experimental results support that nonlinear decision-making is superior to linear decision-making and uniform decision-making. And it further indicates that the DA-GABA-MB mechanism is effective to amplify the conflicting cues and helps UAV complete decision-making task with fewer steps.

#### Application on the UAV obstacle avoidance task

**Figure 3:**
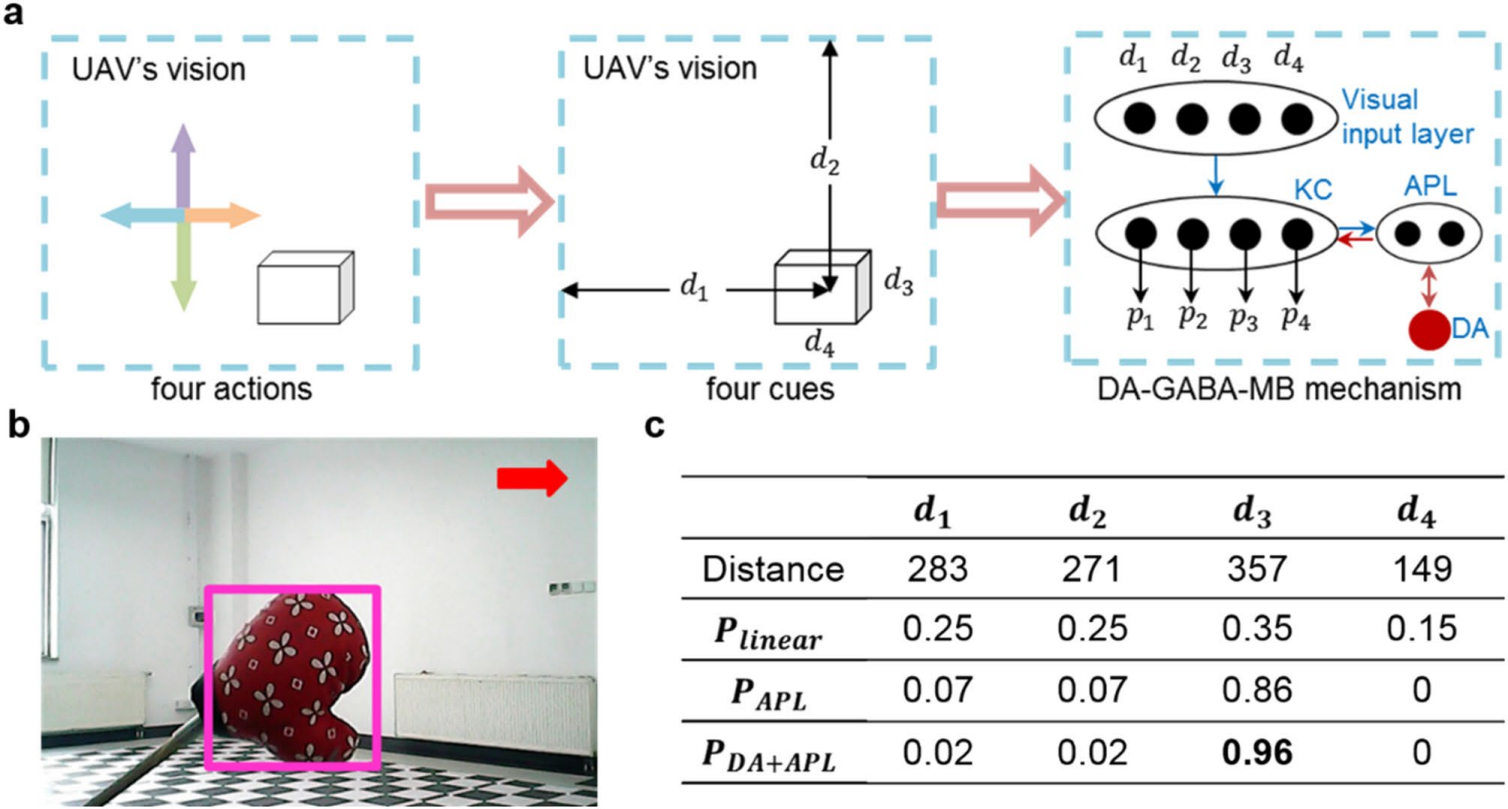
The experimental process and results on the UAV obstacle avoidance task. (**a**) Four conflicting cues learning of the UAV obstacle avoidance task. (**b**) The pink rectangle represents the area of the detected obstacle. The red arrow represents the chosen avoidance action. (**c**) The probabilities of following four cues by linear, only APL and nonlinear manners.

For the UAV obstacle avoidance task, choosing appropriate avoidance behavior is important in order to quickly avoid the danger. In this scenario, the action space contains flying left, up, right and down. The corresponding cues are four distances (left *d*_1_, up *d*_2_, right *d*_3_, down *d*_4_) between the centers of the obstacle and the borders of UAV’s vision respectively, as Fig. 3a shown. The required steps of avoiding obstacle are different for different choices. For example, when the obstacle is on the right side of the UAV, then flying left can quickly bypass obstacle, while flying right is more time-consuming and may lead to potential danger. The four conflicting cues as the input neurons are transmitted to the visual input layer. Then the visual input neurons project to KC layer and the input layer of CC by one-to-one correspondence. We test the effects of linear decision-making, only anterior posterior lateral neurons (APL) decision-making (reciprocal full connections between KC and APL neuron) and nonlinear decision-making manners. The probability of following each cue is $$p_{i} = {\raise0.7ex\hbox{${t_{i} }$} \!\mathord{\left/ {\vphantom {{t_{i} } {\mathop \sum \nolimits_{j}^{n} t_{j} }}}\right.\kern-\nulldelimiterspace} \!\lower0.7ex\hbox{${\mathop \sum \nolimits_{j}^{n} t_{j} }$}}$$, where *t*_*i*_ denotes the number of spikes of cue *i* in KC layer (or input layer of CC for linear circuit), *n* is the number of cues. The inputs are normalized to suit the network’s input (from 1 to 20) of different circuits.

Figure 3b shows an example of the detected obstacle in UAV’s vision, and the obstacle detection algorithm is a stereo vision model, semi-global block matching (SGBM)^20^. After locating the obstacle, four distances *d*_1_, *d*_2_, *d*_3_, *d*_4_ are calculated as the saliency of four cues. It is obvious that the differences among the saliency of four cues are slight. The probabilities of following four cues by different methods are shown in Fig. 3c. For the linear circuit, the probabilities of choosing four choices are similar. By contrast, both the nonlinear circuit and only APL circuit could highlight the most salient cue *d*_3_, then the UAV will fly towards right with a great probability. Compared with only the APL circuit, the nonlinear circuit could maximize the difference of four probability and highlight the most salient cue. To sum up, the DA-GABA-MB mechanism can amplify the difference among the four cues, and make clear-cut decision (with the biggest probability on *d*_3_). While the linear circuit and only APL mechanism perform worse than the DA-GABA-MB mechanism.

### Decision of flying robot UAV

We further apply the DrosDecMa model to the UAV two visual cues learning and the online reversal learning experiments. A DJI MATRIX 100 UAV is used as a test platform. A 2.4 GHz wireless digital video camera (1/4 CCD) is used to acquire visual inputs. We discriminate different visual patterns based on color and shape features. The detailed experimental processes are as follows:

#### Learning two visual cues

**Figure 4:**
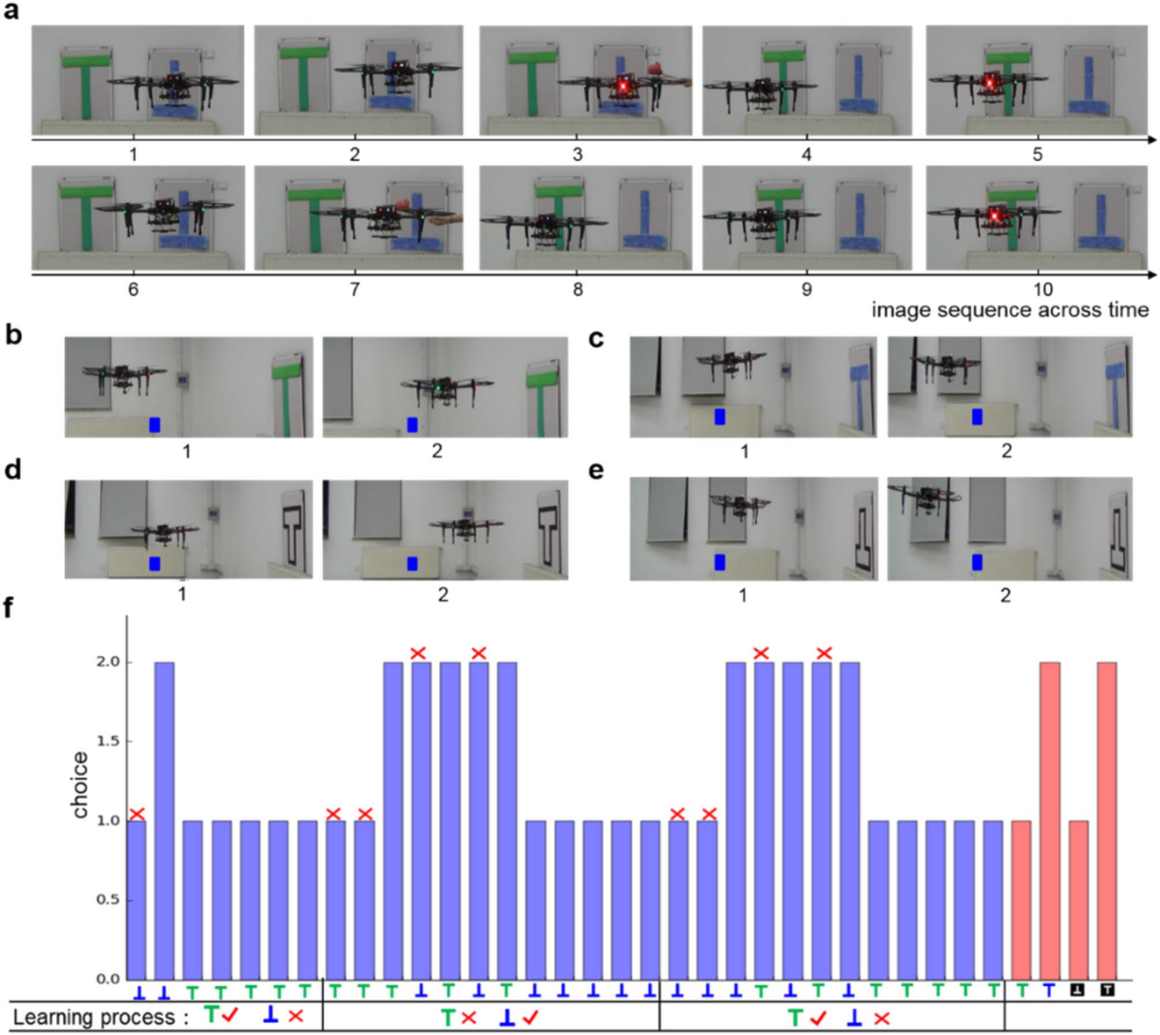
The experimental results of the UAV reinforcement learning and reversal learning. (**a**) The training phase of the UAV two visual cues learning. A red fist is used to simulate heat punishment when the UAV is situated in front of inverted-blue T (image 3 and 7). (**b–e**) The test phase of the UAV two visual cues learning. We test the color learning ability by making choice on upright-green T (**b**) and upright-blue T (**c**). We test the shape learning ability by making choice on upright-white T (d) and inverted-white T (**e**). A blue rectangle is used as the reference object which is stable in different images. (**f**) The experimental results of reversal learning. The learning process contains two times of reverse and the y-axis is the chosen behavior. The blue bars denote the reversal learning process and red bars denote the learning ability on single cue. The red crosses represent incorrect choice and the red check marks represent the correct choice.

The visual input contains upright-green T and inverted-blue T. During the training phase, the output neuron id with the first emitting spike is the chosen behavior. If the model outputs neuron 1, then the UAV keeps still, otherwise, the UAV flies towards another visual pattern. The image sequence of the training phase is shown in Fig. 4a. In the first step, the UAV is situated in front of the inverted-blue T (image 1), and it chooses behavior 1 which means staying here (image 2). Then, it suffers a punishment by projecting a red fist (image 3). The UAV learns the incorrect behavior 1 when confronted with inverted-blue T, and chooses behavior 2 to fly towards upright-green T (image 4). There is no punishment when confronted with upright-green T (image 5) and the UAV has made a correct choice. When situated in upright-green T which the UAV has not learned, it randomly chooses the behavior 2 and flies towards inverted-blue T (image 6). After flying towards inverted-blue T, the UAV suffers fist projection punishment again (image 7) and learns the incorrect choice 2 in upright-green T. Because the UAV has learned the correct behavior 2 in inverted-blue T, it could quickly make choice 2 to fly towards upright-green T (image 8). The UAV has also learned the incorrect behavior 2 in upright-green T, and it chooses behavior 1 to continuously situate in front of the upright-green T (images 9 and 10). As we can see, for each pattern, the UAV can learn the correct choice within one incorrect decision, thus for the whole task of two patterns, at most two incorrect decisions are made before the UVA learns the correct choice.

After training on upright-green T and inverted-blue T, we test the decision-making ability on single cue, which is shown in Fig. 4b–e. Here the behavior 1 represents approaching (flying forward) and behavior 2 represents avoidance (flying backward). We first test the decision-making ability on color learning, and the UAV makes choice between upright-green T and upright-blue T. Figure 4b shows that the UAV approaches upright-green T, while Fig. 4c shows that the UAV avoids upright-blue T. Then, we test the shape learning ability by making choice between upright-white T and inverted-white T. Figure 4d shows that the UAV approaches upright-white T, and Fig. 4e shows that the UAV avoids inverted-white T. These results indicate that the UAV has learned to prefer the upright shape and green color.

#### Online reversal learning

In reversal learning, the UAV first learns to make choice between two patterns with one of them related to punishment. In some cases, the environmental constraints may change, the former rewarded one may become punished, and the former punished one becomes rewarded. The model must be with the ability to capture these changes. Reversal learning is an advanced cognitive ability and we use it to test the adaptability of the model^21^. We design the reversal learning experiments as the following: The DrosDecMa model first learns making choice between upright-green T and inverted-blue T (with punishment). Then after learning the correct choice, we reverse the punishment to upright-green T and inverted-blue T is safe. We try two reversal experiments and summarize the experimental results in Fig. 4f. In the online reversal learning experiment, behavior 1 represents staying in front of the current pattern, and behavior 2 represents avoiding the current pattern and moving towards another pattern.

Firstly, the UAV is situated in front of invert-blue T and chooses behavior 1 to stay, and punishment occurs. Then it flies towards upright-green T and this choice is correct. For the upright-green T, it first tries behavior 1 and it is safe, so the UAV keeps situating in front of the upright-green T. Then we reverse upright-green T to be punished while inverted-blue T is safe. Because the UAV has learned that behavior 1 is correct when facing upright-green T, it will continue choosing behavior 1. In this reversal case, heat punishment happens, and the UAV reduces the probability on choosing behavior 1 in upright-green T. Then, after two punishments, the UAV learns the new rule and chooses behavior 2 to avoid upright-green T and fly towards inverted-blue T. However, because inverted-blue T has been learned to be punished before reversing, the UAV chooses behavior 2. Then the UAV learns the new rule in inverted-blue T and keeps staying in front of it after two punishments. In most cases, the reversal learning ability happens after the UAV makes two incorrect behaviors. As a result, we can conclude that the DrosDecMa model could learn correct choice within one incorrect choice for reinforcement learning, and within two incorrect choices for online reversal learning.

During online reversal learning, it is important to think whether the model still has the ability to learn two cues respectively. Namely, after reversing several times, whether the model could still make the new correct choice? The red bars in Fig. 4f denote the single cue learning ability. After two times of online reversal learning, the UAV has learned the newest rule that upright-green T is safe while inverted-blue T is punished. Then we test color learning by making choice between upright-green T and upright-blue T. The UAV chooses behavior 1 for upright-green T and behavior 2 for upright-blue T, which indicates that the UAV has the ability on color learning after several times of online reversal learning. Experimental results show that shape learning supports the same conclusion.

## Discussion

In this paper, we proposed the DrosDecMa model which simulated the *Drosophila* vision-based linear and nonlinear decision-making circuits from spiking point neuron-level to brain areas-level and behavior level. The decision-making process can freely switch between two distinct neural circuits based on the demand for visual learning tasks. For the linear pathway, we simulated the memory function in CC to make a fast decision. For the nonlinear pathway, we modeled the DA-GABA-MB circuit to implement a gain-gating amplification mechanism. We verified our model on *Drosophila* visual cues learning experiments, the results showed that with the brain-inspired structures and mechanisms, the DrosDecMa model produced similar outputs compared to *Drosophila* behavior experiments in^3,4^. Besides, the application on UAV decision-making tasks showed that the DA-GABA-MB works in a recurrent loop providing a general principle to amplify the difference among multiple conflicting choices and is helpful to make clear-cut decisions quickly.

Specifically, for the influence of CI task, the shape cue learning is not influenced by the change of color's value, while color cue learning depends on the CI. The turning point of CI is 0.4 in^3^, and 0.1 in our case. For two conflicting cues choice, the tendencies of linear and nonlinear circuits in our model are the same as the *Drosophila* behavior experiments^3^, while the CI intersections with *PI* = 0 is different from the *Drosophila* experiment which is 0.8 in^3^, and 1 in our case. The reason is that in our DrosDecMa model, the visual cues are represented as a numeric value, while for biological experiments, the input is not a numeric value, hence leading to some differences.

**Figure 5:**
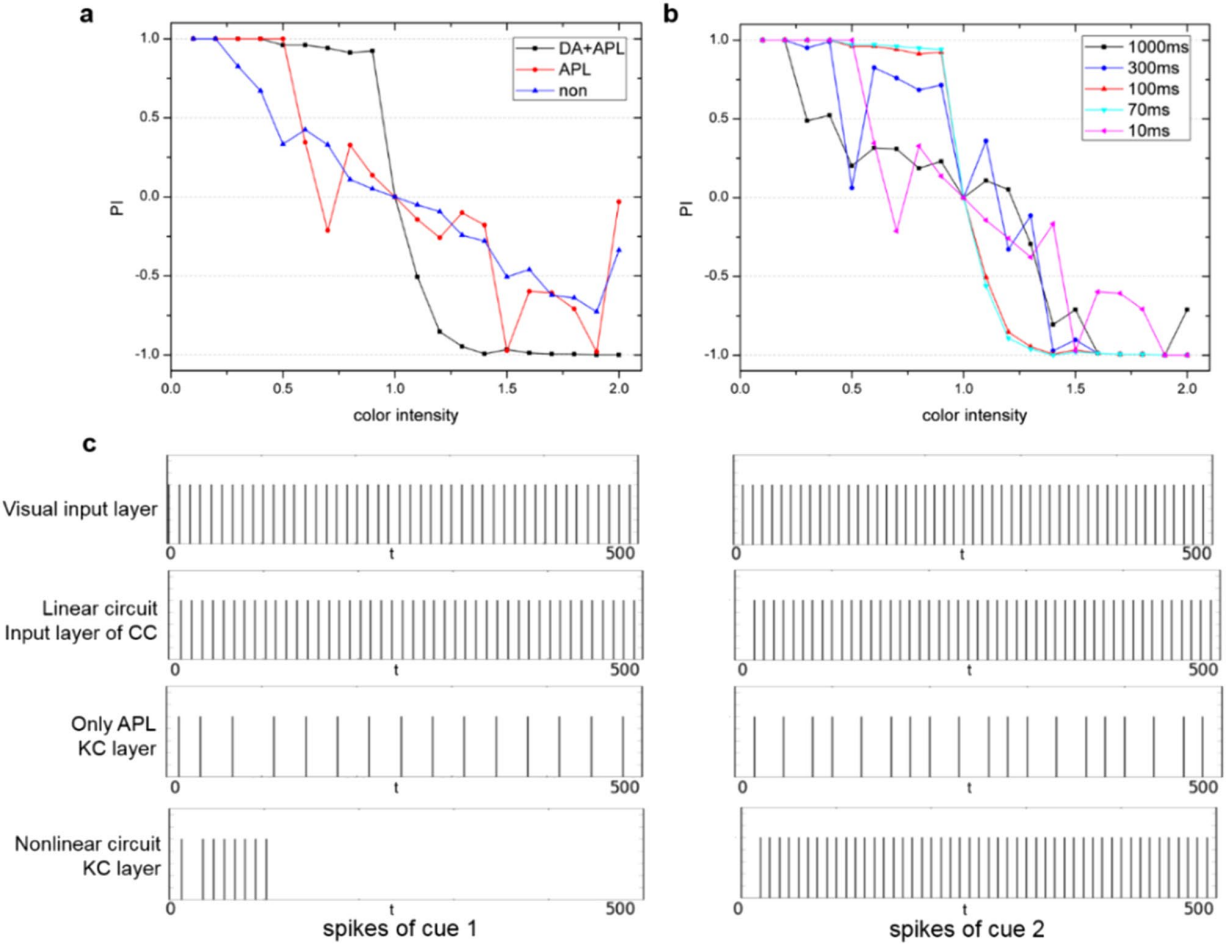
The comparative analysis of DA-GABA-MB mechanism, only APL and non-DA-APL (linear) decision-making manners. (**a**) The experimental results of using both DA and APL, only APL, and non-DA-APL nonlinear decision-making. (**b**) The experimental results on different duration times of DA activity. (**c**) The spike trains of the two cues of the visual input layer, input layer of CC and KC layer under different circuits.

For *Drosophila* visual learning experiments, with the DA-GABA-MB mechanism, the nonlinear circuit is significantly superior to the linear circuit on learning two conflicting visual cues. Now we consider two questions: (1) Why both linear and nonlinear circuits (not just nonlinear circuit) are needed in *Drosophila* brain? When confronted with non-conflicting cues, the linear circuit with simple connection structure is enough to solve vision-based learning. Only when there exist conflicting cues, the linear circuit could not perform the clear-cut decision, then the nonlinear circuit with DA-GABA-MB mechanism will be opened to solve this situation. Adaptively switching between linear and nonlinear circuits is the embodiment of brain economics. For the UAV reinforcement learning tasks introduced in the Results section, the linear circuit is enough to achieve vision-based learning, and for this kind of task, the nonlinear circuit will not be opened. While in the case of multiple conflicting choices, the nonlinear circuit outperforms the linear circuit and we apply it to the UAV multiple conflicting decision-making tasks. As a result, the switching between linear and nonlinear circuits depends on the demand of the task. (2) Why the nonlinear circuit performs better than linear circuit when confronted with multiple conflicting options? The training phase of nonlinear and linear circuits are the same, and the choice phase does not change the connection weights between input and output neurons at all. The major difference between nonlinear and linear circuits is the DA-GABA-MB mechanism. It is the core reason why the nonlinear circuit could perform the ambiguous decision-making task well, while the linear circuit could not. We eliminate the effects of DA or APL in our model to test the result of decision-making respectively, as shown in Fig. 5a. When we eliminate both the effects of DA and APL on MB, the result is shown as the blue line in Fig. 5a, which is the same as the curve of the linear circuit. When eliminating the effect of DA, then only APL continuously inhibits KC, the result is shown in the red line in Fig. 5a, which is unstable for decision-making. The experimental results indicate that DA and APL collectively and interactively play important gain-gating roles in nonlinear decision-making^4,6^.

In the proposed model, the duration time of DA activity is 100 ms. It is essential to analyze and discuss how the duration time of DA activity influences the result of nonlinear decision-making. We test the results of different duration times on nonlinear decision-making as shown in Fig. 5b. All results with duration time are 1000 ms, 300 ms and 10 ms are worse than 100 ms and 70 ms, which indicates that the decision would be failed when the duration time of DA activity is too long or too short. The difference between the results of 100 ms and 70 ms is relatively not obvious, which indicates the good generalization for different DA duration time given an appropriate time slot.

We further analyze the effect of DA-GABA-MB mechanism on nonlinear circuit. For nonlinear circuit, the DA-GABA-MB mechanism can amplify the slight difference between two conflicting cues. We take two conflicting cues (cue 1 is equal to 9, and cue 2 is equal to 10) as an example, the spikes of the visual input layer are shown in Fig. 5c. Then, we compare the spikes of the input layer of CC for linear circuit, the KC layer for only APL circuit, and nonlinear circuit. Here we only show the spikes during the first 500 ms. For the linear circuit, there is only a slight difference between the spikes of the two cues of the CC’s input layer. For the circuit only with APL’s inhibition, two cues are both inhibited by APL and the numbers of two cues’ spikes are reduced but the difference between them is not amplified. For nonlinear circuit, during the first 100 ms, where DA is activated, the number of spikes for the more salient cue is bigger than the weaker one. After that, APL continuously inhibits the weaker cue. In this way, DA facilitates the salient cue pass and APL suppresses the weaker cue pass.

**Figure 6:**
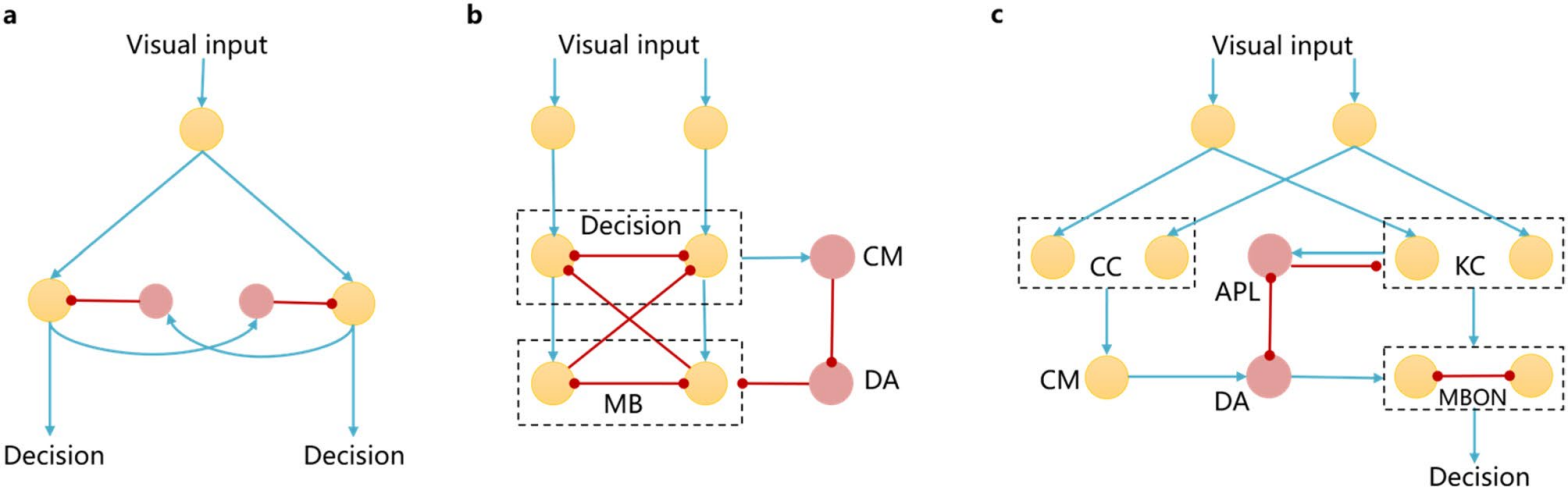
The network architecture in^7^ (**a**)^9^, (**b**) and our method (**c**). The red circle represents inhibited neurons. The orange circle represents excited neurons. The red line represents inhibited connections. The blue line represents excited connections. The CM is the conflict monitor.

Previous related studies on the construction of *Drosophila* decision-making neural circuits can be divided into two categories. Wei et al.^7^ and ^8^ mainly focused on the connections and interactions among excitatory and inhibitory spiking neurons during the training phase. The network circuit is shown in Fig. 6a. Punishment regulates learning by inhibiting input neurons and activating output neurons. Wei et al.^8^ considered conflicting cues learning compared with^7^, but the network structure of nonlinear decision-making has no change. For conflicting cues learning, the input cues are encoded by a linear or nonlinear probability form, and then transmit to the decision-making network. The decision-making network is the integration of multiple simple circuits, when confronted with the unseen condition, the network needs to be expanded. Besides, the nonlinear shape is based on the nonlinear input pattern, not the effect of neural circuit. This paper makes sure that the visual input is the same both in the linear and nonlinear decision-making circuit, and only the different neural circuit leads to different results. Thus, the nonlinear decision-making ability is attributed to the DA-GABA-MB circuit, which is shown in Fig. 6c.

Cai et al.^9^ and Wu and Guo^10^ mainly focus on the decision-making process on conflicting cues. Cai et al.^9^ considered that nonlinear decision-making is related to the dopamine modulations on MB. Here, dopaminergic neurons send tonic inhibitory inputs to MB, suppressing its connection to the decision-making layer in normal situations. When conflicting cues are observed, conflict monitor inhibits dopaminergic neurons and consequently the gate for the MB output to the decision-making layer is opened. MB receives the input from decision-making layer and in return lateral inhibits the other choice in the decision-making layer. The network architecture is shown in Fig. 6b. This method considers partial decision-making mechanism in *Drosophila*, but lacks the complex MB related decision-making circuit. Wu and Guo^10^ simulated the nonlinear circuit by a raised dopamine level to enhance the lateral inhibition between decision-making units. Cai et al.^9^ and Wu and Guo^10^ are focus on mathematical calculations, which have no spike information transmission. To sum up, the existing models do not reflect the actual working mechanism of *Drosophila* decision-making system. This paper simulates the CC for linear circuit and DA-GABA-MB loop for nonlinear circuit respectively, which shows more biologically plausible.

**Figure 7:**
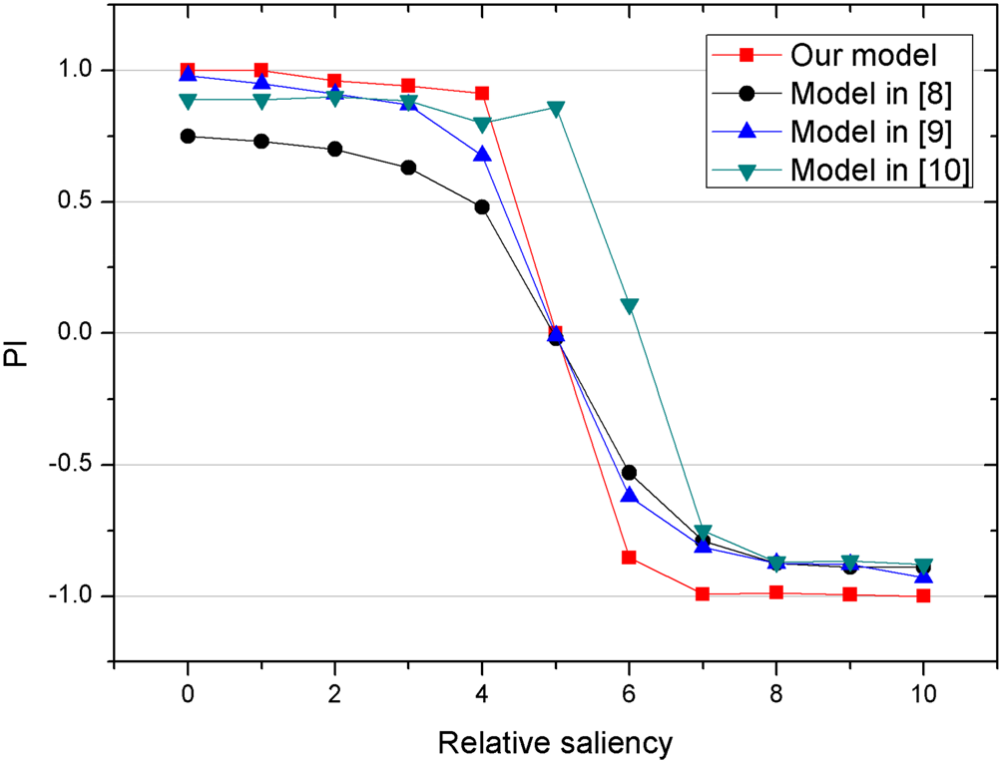
The comparative results of our model with other biologically inspired models on conflicting cues learning tasks.

For conflicting cues learning experiments, we do a deep comparison and analysis of our proposed model and the existing models (including^8–10^). To make sure that the comparison is feasible, we unify the curve of nonlinear decision-making results of different models in the same dimension space and mainly observe the change trend of PI. By changing the color intensity of cues, a Boltzmann fit of PI produced when using sigmoid-shaped color-shape selection curve as input, and the changing of PI is depicted in Fig. 7 (black line)^8^. In the presence of conflicting cues, Cai et al.^9^ provides two biased inputs $I_{b}^{1}$ and $I_{b}^{2}$ as the input cues of decision-making layer. The two bias inputs represent the two competitive choices induced by two conflicting cues. Then, one bias is fixed while the other is changed, and simulations are repeated under different distances between two bias $I_{b}^{1} - I_{b}^{2}$. The blue line in Fig. 7 shows the change of PI with different $I_{b}^{1} - I_{b}^{2}$. The conflicting cues learning experiments in Wu and Guo^10^ focus on the color-position dilemma. Δ is used to represent the position saliency of visual targets. By changing Δ and repeating the choice task, the change of PI is obtained and shown in the green line of Fig. 7. Actually, different *CI*, $I_{b}^{1} - I_{b}^{2}$, and Δ indicate the relative saliency of two visual cues. For different models, PI exhibits a sigmoid shape consistently, which indicates the nonlinear decision-making ability of these models. Comparing our method (the red line in Fig. 7) with other models, our method can amplify the small difference more clearly and contributes to clearer winner-takes-all decision.

Thus, the DrosDecMa model captures the necessary biological characteristics of *Drosophila* vision-based linear and nonlinear decision-making circuits and mechanisms. It can successfully describe and predict the experimental findings in *Drosophila*. Furthermore, the UAV can quickly make clear-cut decisions among multiple conflicting choices by introducing the DA-GABA-MB mechanism. It is our first preliminary attempt to bring Brain Science data and Brain-inspired neural network design together. People will ask, will such a study help us understand how high-level brain works as a whole? From Darwinian evolution-selection point of view, the MB in insect brains may represent an evolutionary primitive brain structure homologous in form and function to structures in the high-level brains^22–24^. It is worth mentioning that it has been revealed surprising common circuit design in fly and mammalian motion vision systems only recently while at first glance, the eyes of mammals and compound eyes of insects do not seem to have much in common^25^. It is well known that in human vision are multi-stable percepts such as the Necker cube or Rubin's vase. It has been demonstrated recently that also *Drosophila* may experience sensory ambiguity and multi-stable perception^26^.

Primate decision-making can also be divided into linear (perceptual decisions) and nonlinear decision-making (value-based decision-making)^27^. Dopamine, as a prediction error in reinforcement learning, and as well as GABAergic system also plays a crucial role in value-based decision-making in primate^28^. So, we believe that the elucidation of the fly’s decision-making algorithm may provide some insight into complex decision making in the high-level brains. Thus, it encourages us to further explore the relationship and differences between *Drosophila*’s and primate’s brain for their decision-making mechanisms, and in this way to build-up a brain-inspired intelligence model.

## Methods

### The neuroanatomy of *Drosophila* decision-making system

Linear circuit and non-linear circuit work together contributing to the *Drosophila* decision-making system. The linear circuit is the fast circuit for simple perceptual decision-making. When *Drosophila* confronts a shape-color dilemma, the nonlinear circuit exhibits clear-cut decision by amplifying the difference between the slight saliency of two conflicting cues^4,6^. The *Drosophila* can adaptively switch between the two neural circuits based on the demand of vision-based learning tasks. Here, we simplify the process of visual information processing and action execution. A series of behavior experiments have evidenced that five visual features including color, shape, size, vertical position, and elevation can be recognized by *Drosophila*^29^. The color and shape information are firstly extracted by the compound eye of *Drosophila*^6,30^. The inhibitory connections among the fly’s visual system are useful for visual selective attention and filtering specific cue to the central nervous system (e.g. CC and MB)^31^.

Linear decision-making circuit. The main brain area of the visual linear decision-making circuit in *Drosophila* brain is CC. Short-term memory is localized in fanshaped body of CC^9^. Pan et al. evidenced that ellipsoid body of CC is also involved in visual memory^32^. CC is also in charge of flying because it is the area where decision is made first. After the decision is made there, it will be projected to the motor area^1^. The linear circuit is almost enough to make choice when the visual input is simple and non-conflicting. When confronted with conflicting cues, the linear circuit could not make clear choice, and nonlinear circuit will be opened to make clear-cut decisions^9^.

Nonlinear decision-making circuit. This circuit mainly depends on MB and DA system^3,4,10^. MB is the most important structure in *Drosophila*, which is constituted mainly by about 2000 KC^33,34^. When confronted with conflicting visual cues, DA-MB circuit could provide a gain-gating mechanism to enhance the relative salient cue and suppress the less salient cue, thereby modulates the decision-making^4,6^. Conflicting visual cues induce a phasic response of dopaminergic neurons and then result in an increase of dopamine levels in MB, and such an increase may sustain 70–100 ms^35^. On the other hand, there is a pair of APL GABAergic interneurons, which innervate the peduncle, whole lobes (output area) and calyx (input area) of MB, have been found both pre synapses and post synapses at all areas. It has been demonstrated in locusts the existence of a normalizing negative-feedback loop connection between the MB and a "giant" non-spiking GABAergic inhibitory interneuron (equivalent to APL in fly)^36^. Using the single cell RNA-sequencing technology, we discovered the reciprocal connections between KC and APL neurons that KC could activate the APL neurons, and in turn the APL neurons release the GABA transmitter to inhibit the activity of KC^11^. Further, we identified a new synaptic connection from the DA neurons to the APL neurons and characterized an inhibitory dopamine signal in this DA-to-APL synapse^12^. Thus, the increase of dopamine inhibits APL's activity which is mediated by the Dopamine 2-like receptor (DD2R). In this way, DA facilitates the relatively salient cue pass until the DA's level falls, and after that, APL continuously inhibits visual inputs^37^. Together the DA-GABA-MB circuit mechanism provides a gain-gating mechanism for decisive and sharp winner-takes-all decision-making^6^.

### The DrosDecMa model

The proposed DrosDecMa model simulates the linear and nonlinear decision-making circuits in *Drosophila* from spiking point neuron-level to brain areas-level, and is with similar behavior outputs compared to *Drosophila*. In this section, we will introduce the detailed model implementation process. SNN is considered as the third generation of Artificial Neural Networks^38^. It encodes the information in spike trains instead of spike rates as in the Artificial Neural Networks^39^. That makes SNN more biologically plausible, and more computationally feasible for modeling cognitive functions^40,41^. We use Brian simulator^42,43^ to model the DrosDecMa Spiking Neural Network. Because the leaky integrate-and-fire (LIF) neuron model^44^ is simple and computationally efficient, this paper uses LIF neuron model to build SNN and the calculation formula is shown in Eq. (2), where *v*(*t*) represents the membrane potential at time *t*, *τ*_*m*_ is the membrane time constant and *R* is the membrane resistance. When the membrane potential *v*(*t*) reaches a certain threshold *v*_*th*_, it is instantaneously reset to a lower value *v*_*r*_. Here, we set $v_{th} = 0.1, v_{r} = 0, \tau_{m} = 20$.

2 $$\tau_{m} \frac{{d_{v} }}{{d_{t} }} = - v\left( t \right) + RI\left( t \right)$$

In *Drosophila* visual learning experiment, two visual patterns are provided at the same time, and *Drosophila* needs to make a decision on one of the two patterns, which is called current visual input. Each visual pattern has two cues, namely shape and color cues, such as upright-green T and inverted-blue T. Here, we use five neurons to represents the visual input. The first three neurons represent R, G, B color space respectively, and the last two neurons represent upright or inverted T. For example, [0, 10, 0, 10, 0] represents upright-green T and [0, 0, 10, 0, 10] represents inverted-blue T. The value 10 represents the strength or the saliency of color and shape cues. The neurons in the visual system are used to convert external stimuli to spiking trains. When confronted with two candidate visual patterns, *Drosophila* needs to make choice to fly towards one of the two visual patterns. For two competitive visual patterns, *Drosophila* visual system can automatically filter out noise information and selectively pay attention to salient visual cue by simple subtraction of two input visual patterns. Every time the current visual input is directly projected to the visual system, and the other visual pattern subtracts this current visual input by one-to-one inhibition. In this way, when confronted with upright-white T and inverted-white T, the color cue (white) is the noise because the color is the same in two visual patterns, and the visual system could filter out the color cue and only allow shape cue to pass.

The *Drosophila* visual learning experiment contains two phases: namely, the training phase and the choice phase. The detailed network architecture, learning and working mechanism of the training phase and choice phase are introduced as follows:

#### Training phase

**Figure 8:**
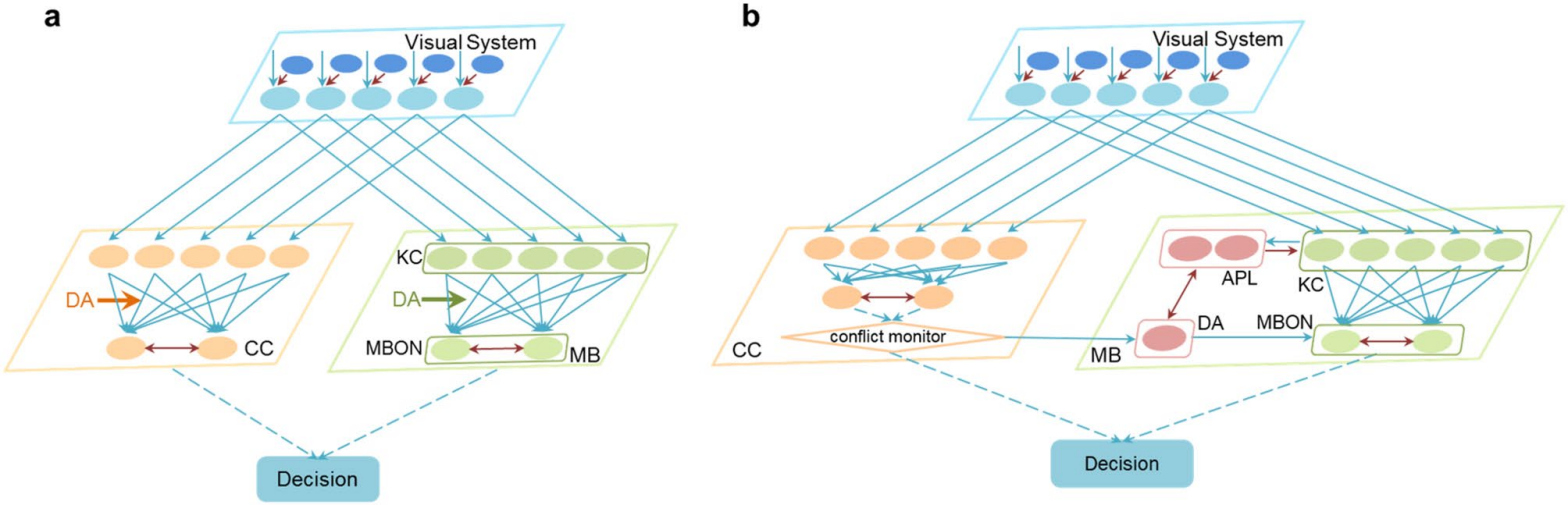
The training phase (**a**) and the choice phase (**b**) of the DrosDecMa network architecture. The blue arrows represent excitatory connections, and the red ones represent inhibitory connections.

The training phase learns the safe visual pattern between two visual patterns with heat punishment on one specific visual pattern^4^. Then the model needs to learn the safe visual pattern and the corresponding shape and color cues. Visual input is projected to the linear circuit and nonlinear circuit at the same time, and the training phase of two circuits are the same. The basic architecture is shown in Fig. 8a. Here, the orange areas represent the main brain regions (CC) of the linear circuit, and the green areas represent the main brain regions (MB) of the nonlinear circuit. Every time we input one visual pattern and the other as the inhibitory input for filtering out the noise features. The visual information then projects to the input layer of CC and the KC layer of MB by one-to-one correspondence. As a result, the number of neurons in visual system is 5, and the number of neurons in KC and input layer of CC are 5. In the CC, we design the linear decision-making network with two layers which are corresponding to the input layer and output behavior layer respectively. The connections between the input layer and the output layer in CC are full connections. The output behavior contains behavior 1 (approaching) and behavior 2 (avoidance). In MB, the KC receives visual cues from the visual system, and has full connections with mushroom body output neuron (MBON) to produce output behavior^34,45^. The output behavior also contains behavior 1 (approaching) and 2 (avoidance). The mutual inhibition exists between two output neurons of CC and MBONs of MB^46^.

For two visual patterns, one is safe and the other is punished. The training phase separately learns each visual pattern for 1 s. When given visual input, the output neuron id with the first emitting spike is the chosen behavior. The punishment occurs when choosing behavior 1 under punishment-related visual input and choosing behavior 2 under safe visual input. Then dopamine neurons modulate the learning process of both linear and nonlinear circuits. This paper combines dopamine regulation and STDP mechanism to modulate the network’s training process, which shows more biologically plausible.

#### Synaptic plasticity

STDP is widely used as an unsupervised learning principle for SNN. It is used to learn connection weights according to the relative time between spikes of presynaptic and postsynaptic neurons. The modulation rule is: If the postsynaptic neuron fires a few milliseconds after the presynaptic neuron, the connection between the neurons will be strengthened, otherwise, the connection will be weakened^47^. The update function is shown in Eq. (3), where *A*_+_ and *A*_−_ are learning rates, *τ*_+_ and *τ*_−_ are time constant, and Δ*t*_*i*_ is the delay time from presynaptic spike to postsynaptic spike. Here, we set $A_{ + } = 0.925, A_{ - } = 0.9, \tau_{ - } = \tau_{ + } = 20$.

3 $$\Delta w_{j} = \left\{ {\begin{array}{*{20}l} { - A_{ - } e^{{\left( { - \Delta t_{i} /\tau_{ - } } \right)}} } \hfill & {\quad \Delta t_{i} > 0} \hfill \\ {A_{ + } e^{{\left( {\Delta t_{i} /\tau_{ + } } \right)}} } \hfill & {\quad \Delta t_{i} < 0} \hfill \\ \end{array} } \right.$$

Because STDP is triggered when the postsynaptic neuron sends out spike, it is considered as a local, instantaneous modulation mechanism. During the training phase, STDP happens in all the connections of the training network.

#### Dopamine regulation

The training phase is a reinforcement learning process to learn correct behavior, and heat punishment is the reinforcement signal. Dopamine modulates the learning process because it is considered as carrying punishment signal^29^. Dopamine-induced synaptic plasticity contributes to the formation of aversive memories in insect. Experiments show that DA could perform different functions, such as saliency sensitive, reward sensitive and punishment sensitive^48,49^. Here, punishment-related DA performs as a global neuromodulator to regulate the specific connections. Compared with the local STDP mechanism, DA works when the punishment occurred, and DA only makes a difference in the specific connections which are leading to the punishment. Punishment-related DA increases transiently in the MBs in response to aversive stimulation^50^, and the phasic dopamine regulates the change of synaptic activity in MB. Two types of dopamine receptors, D1 and D2, that are instrumental in responding to reward and punishment, respectively. The D2 receptors induce long term depression (LTD) on postsynaptic neurons, and D1 receptors induce long term potentiation (LTP) on postsynaptic neurons^51^. Thus, heat punishment triggers the increase of dopamine, and then inhibit the specific synapses between KC and MBON through inhibitory dopamine receptor. In this way, punishment-DA makes it harder for postsynaptic neurons to fire.

Phasic dopamine increases when punishment occurs, and then exponentially decays to 0. We assume that:

4 $$DA\left( t \right) = DA_{peak} *e^{{ - {\raise0.7ex\hbox{${\left( {t - t_{pun} } \right)}$} \!\mathord{\left/ {\vphantom {{\left( {t - t_{pun} } \right)} {\tau_{t} }}}\right.\kern-\nulldelimiterspace} \!\lower0.7ex\hbox{${\tau_{t} }$}}}}$$

where *DA*(*t*) represents the dopamine concentration at the given time, *t* represents current time, *t*_*pun*_ represents the moment dopamine increases, *DA*_*peak*_ is the maximum concentration of phasic dopamine, and *τ*_*t*_ is the exponential constant. Here, we set $DA_{peak} = 10, \tau_{t} = 2$.

Then the weight update formula is:

5 $$\Delta w = - DA\left( t \right)$$

here *w* is the connection weights between visual pattern activated input neuron and chosen output neuron in CC, and between visual pattern activated KC and chosen MBON in MB).

Because the learning process is the same in CC and MB, the connection weights between the input layer and the output layer of CC and the connection weights between KC and MBON layer of MB which will be used in the choice phase are the same.

#### Choice phase

After the training phase, the choice phase is used to test the decision-making ability. The choice phase directly makes preferred choice between two visual patterns. The visual system transmits the filtered current visual input to decision-making circuit, and then output the chosen behavior. *Drosophila* visual learning experiment contains two behavior choices, staying or approaching current visual input and approaching the other visual pattern. To describe the behavior choice ability, the proposed SNN model is performed 2 s on one current visual pattern, and we count the chosen behavior which is the output neuron id with the first emitting spike to calculate PI. From the calculation formula of PI shown in Eq. (1), *PI* = 0 means the time of choosing two visual patterns is the same, which indicates that the model could not make clear decision between the two visual patterns. *PI* > 0 indicates that the model almost approaches current visual input, while *PI* < 0 indicates that the model almost avoids current visual input, and the closer that the absolute value to 1, the clearer choice the model can make. The network architecture of the choice phase is shown in Fig. 8b. The differences of network architecture between the choice phase and training phase are the conflict monitor in linear circuit and the DA-APL-KC loop in nonlinear circuit.

The choice phase needs conflict monitor to determine which circuit (linear or nonlinear) should be followed. CC and MB function as action selection, and fast decision is made in CC first. Thus, a conflict monitor is designed as the output of the linear circuit, and also as a switch of the nonlinear circuit. If the linear circuit is enough to make clear decision, then there is no need to activate the nonlinear circuit. Only when the linear circuit could not make a clear choice, will the nonlinear circuit be activated. The conflict monitor rules can be summarized as the following: (1) Single visual cue only needs linear circuit. (2) Two non-conflicting cues need linear circuit. (3) Two conflicting cues need nonlinear circuit. Conflicting occurs when the two cues correspond to different correct behavior, such as one of two cues is safe while the other is punished. In this case, the output neurons of the linear circuit have similar spiking responses so that it could not make a clear choice on the visual input with conflicting cues. The memory of the correct choice has been saved in the connections weight in CC. And for correct choice neuron, the connect weight is large, while the connect weight is small for the incorrect choice. For each choice (output neuron of the linear circuit), we sum the connection weights between input cue and output choice to judge whether a conflict exists. Here, we define *s*_1_ as the sum of connection weight between input layer and neuron 1 of output layer in CC, and *s*_2_ as the sum of connection weight between input layer and neuron 2 of output layer in CC. If the difference between *s*_1_ and *s*_2_ is large enough (beyond the threshold *th*), then the linear circuit could make clear choice. In this paper, *th* is set to 3. The conflict monitor function is shown in Eq. (6).

6 $$f = \left\{ {\begin{array}{*{20}l} {linear} \hfill & {\quad \left| {s_{1} - s_{2} } \right| > th} \hfill \\ {non - linear} \hfill & {\quad \left| {s_{1} - s_{2} } \right| \le th} \hfill \\ \end{array} } \right.$$

All the parameters used in the proposed model are summarized in Table 1.

**Table 1:** The description of all the parameters in the proposed model.

*Neuron model*	
*v*	Membrane potential
*τ*_*m*_	Membrane time constant
*R*	Membrane resistance
*v*_*th*_	Threshold value
*v*_*r*_	Reset value
*STDP mechanism*	
*A*_+_	Learning rates of LTP
*A*_−_	Learning rates of LTD
*τ*_+_	Time constant of LTP
*τ*_−_	Time constant of LTD
Δ*t*_*i*_	Delay time from presynaptic spike to postsynaptic spike
*DA regulation*	
*DA*	The concentration of dopamine
*DA*_*peak*_	The maximum concentration of phasic dopamine
*τ*_*t*_	Exponential constant
*t*_*pun*_	The moment dopamine Increases
*Conflict monitor*	
*s*_1_	The sum of connection weight of behavior 1
*s*_2_	The sum of connection weight of behavior 2
*th*	The threshold of conflict monitor

When conflicting cues are detected, the nonlinear circuit is opened and the visual cues enter MB. The only difference between the nonlinear circuit and the linear circuit is the DA-GABA-MB mechanism, which contains the interactive suppression between DA and APL, and the disinhibition effect on KC. Here we provide one neuron to represent the DA, and two neurons to represent APL. The APL neurons are activated by the KC and send negative feedback to the KC, and this feedback loop is essential for sparse coding in the MB^52,53^. GABAergic neurons (APL) also receive suppressive signals directly from DA through inhibitory dopamine receptor, DD2R. This implies that DA has a disinhibition effect on KC. Besides, DA receives the suppressive signals from APL, and has excitatory connections with MBON. STDP happens in visual system-KC, KC-MBON, APL-KC, APL-DA, DA-MBON connections.

The DA-GABA-MB circuit contributes to gain-gating mechanism for quick winner-takes-all decision-making^4,6^ and the basic process is as the following: When conflicting cues occurred, the DA neuron will be activated for 100 ms. The activation of DA raises the increase of GABA receptor in APL neurons^12^. The increase of DA inhibits APL's activity and then has a disinhibition effect on KC^46^. In this way, DA first facilitates the activation of relatively salient cue and the corresponding behavior choice until the DA level falls, and then APL continuously inhibits the KC^12,37^. Finally, valence encoded by MBON ensemble biases memory–based action selection. Because of the activation of DA, the relatively salient cue passes more in KC layer, and also the corresponding choice in MBON layer is more. Then the more salient cue related connection weights between the visual system and KC (and between KC and MBON) will be strengthened based on STDP, while the weights for the less salient cue are smaller. Then, after the DA activation, APL inhibits KC and the less salient cue will be inhibited more than the salient cues. As a result, the DA-GABA-MB mechanism contributes a quick discrimination of a slight difference between two cues' saliency.

## Conclusion

This paper proposed a DrosDecMa spiking neural network model which mainly simulated the *Drosophila* vision-based linear and nonlinear decision-making circuits. A conflict monitor is used to freely switch between two distinct neural circuits based on the demand of visual learning tasks. For the linear circuit, we simulated the memory function in CC to make a fast decision. For the nonlinear circuit, we modeled the DA-GABA-MB mechanism from both connectome and functional perspectives. With the brain-inspired structures and mechanisms, the DrosDecMa model could produce similar outputs compared to *Drosophila* visual cues learning experiments. Besides, the application on UAV decision-making tasks showed that the DA-GABA-MB works in a recurrent loop providing a simple but general principle to amplify the difference among multiple conflicting choices and help the UAV complete the task faster.
